# Progress towards malaria control targets in relation to national malaria programme funding

**DOI:** 10.1186/1475-2875-12-18

**Published:** 2013-01-14

**Authors:** Eline L Korenromp, Mehran Hosseini, Robert D Newman, Richard E Cibulskis

**Affiliations:** 1 Department of Public Health, Erasmus MC, University Medical Center Rotterdam, Rotterdam, The Netherlands; 2 The Global Fund to Fight AIDS, Tuberculosis and Malaria, Geneva, Switzerland; 3 Global Malaria Programme, World Health Organization, Geneva, Switzerland

**Keywords:** Malaria/mortality, Malaria/prevention and control, Child health, Programme impact, Financing, Health resources, Investments, Millennium Development Goals, Resource-poor countries

## Abstract

**Background:**

Malaria control has been dramatically scaled up the past decade, mainly thanks to increasing international donor financing since 2003. This study assessed progress up to 2010 towards global malaria impact targets, in relation to Global Fund, other donor and domestic malaria programme financing over 2003 to 2009.

**Methods:**

Assessments used domestic malaria financing reported by national programmes, and Global Fund/OECD data on donor financing for 90 endemic low- and middle-income countries, WHO estimates of households owning one or more insecticide-treated mosquito net (ITN) for countries in sub-Saharan Africa, and WHO-estimated malaria case incidence and deaths in countries outside sub-Saharan Africa.

**Results:**

Global Fund and other donor funding is concentrated in a subset of the highest endemic African countries. Outside Africa, donor funding is concentrated in those countries with highest malaria mortality and case incidence rates over the years 2000 to 2003. ITN coverage in 2010 in Africa, and declines in case and death rates per person at risk over 2004 to 2010 outside Africa, were greatest in countries with highest donor funding per person at risk, and smallest in countries with lowest donor malaria funding per person at risk. Outside Africa, all-source malaria programme funding over 2003 to 2009 per case averted ($56-5,749) or per death averted ($58,000-3,900,000) over 2004 to 2010 tended to be lower (more favourable) in countries with higher donor malaria funding per person at risk.

**Conclusions:**

Increases in malaria programme funding are associated with accelerated progress towards malaria control targets. Associations between programme funding per person at risk and ITN coverage increases and declines in case and death rates suggest opportunities to maximize the impact of donor funding, by strategic re-allocation to countries with highest continued need.

## Background

With support from development partners, low- and middle-income countries have intensified efforts to meet intervention coverage and impact targets of the Millennium Development Goals (MDGs) [1], the World Health Assembly (WHA) and the Roll Back Malaria (RBM) Partnership [2] (Table 1). International funding for malaria control increased from US$149 million in 2000 to approximately US$1.66 billion in 2011 [3,4]. The Global Fund has been a major contributor to this scale-up, covering an estimated 40% of international assistance for malaria from 2003 to 2008, and 63% of international malaria funding in the year 2010 alone [3,5]. Between 2002 and 2011, Global Fund-supported malaria programmes delivered 230 million insecticide-treated nets (ITNs) and 230 million malaria treatments across 84 low- and middle-income countries [5,6]. This external funding is complemented by the United States President’s Malaria Initiative (PMI), which, since 2005, has been a significant donor for malaria control programmes in 19 countries in Africa and the Greater Mekong sub-region in Asia.

**Table 1:** Malaria targets in the Millennium Development Goals and of the World Health Assembly and Roll Back Malaria Partnership

	
MDG 6 − Combat HIV/AIDS, malaria and other diseases [1]:	
• Target 6c: By 2015, have halted and begun to reverse the incidence of malaria and other major diseases	
• Indicator 6.6: Incidence and death rates associated with malaria	
• Indicator 6.7: Proportion of children under-five sleeping under an insecticide-treated bed net	
MDG 4 − Reduce child mortality [1]:	
• Target 4a: Reduce by two-thirds, between 1990 and 2015, the under-five mortality rate.	
World Health Assembly [43]:	
• Reduce malaria cases by 75% from 2000 to 2015	
• Reduce malaria-related deaths by 75% from 2000 to 2015.	
Roll Back Malaria Partnership, June 2011 [2,3]:	
• Objective 1: Near-zero malaria deaths by 2015.	
• Objective 2: Reduce malaria cases by 75%, from 2000 to 2015.	
• Objective 3: Eliminate malaria by 2015 in 10 new countries and in the WHO European Region.	
• Target 2.2 Sustain universal access to and utilization of prevention measures: By 2015 and beyond, all countries sustain universal access to and utilization of an appropriate package of preventive interventions.	
• Indicator: Proportion of households with at least one ITN.	

As a consequence of increased coverage with ITNs and artemisinin-based combination therapy (ACT), a growing number of countries have reported decreasing numbers of confirmed malaria cases and/or malaria-attributed hospital admissions and deaths since 2000; globally, malaria mortality has fallen by an estimated 25% [3,7].

RBM and WHO estimate the financing need for effective malaria control to exceed US$5 billion every year through 2015; yet even at its 2011 peak, global funding available for malaria remained far below this need, with international disbursement totalling US$2 billion and domestic financing a lower amount [3,8,9]. To allocate available funding effectively and maximize the impact of investments, it is critical to understand how and where funding is advancing programme success in terms of population coverage achieved as well as health impact [3,9].

This study compares changes in the proportion of households owning at least one ITN (for sub-Saharan Africa countries) and in malaria cases and deaths (for countries outside Africa) with levels of malaria programme funding (from the Global Fund, other donors and domestic governments) available to low- and middle-income countries over the period 2003 to 2010. Findings are discussed with a view towards informing donor investment strategies and allocation policies.

## Methods

### Malaria programme financing

Malaria programme funding in each endemic country was expressed as the cumulative funding between 2003 (the first year of Global Fund malaria disbursements) and December 2009, in current US dollars. To assess the effects of funding on progress with malaria control, given that available estimates on malaria cases and deaths extended to 2010, analysis of funding data was capped at December 2009, assuming a typical lag of one year to in-country spending, programme implementation [10] and ensuing impact on reducing malaria cases and deaths.

Allocations from the Global Fund were taken as disbursements through malaria grants [5,11]. Other-donor gross disbursements included those from the USA President’s Malaria Initiative [12], World Bank [13], and other bilateral and multilateral donors as reported through the Organization for Economic Cooperation and Development (OECD) Development Assistance Committee (DAC)’s Creditor Reporting System [14]. Domestic financing data was taken from reports by National Malaria Control Programmes to WHO through the annual World Malaria Report questionnaire [3]. Government funding is expressed as programme expenditures, or as budgets when a programme had not reported government expenditures [15].

Total country funding was converted into funding *per-capita* using national population sizes [16], and into funding per person at risk using WHO estimates of national populations at risk [3]. For analyses, countries were stratified into three groups, according to their level of funding from all donors (Global Fund and other donors combined) cumulated over 2003 to 2009 per person at risk (Additional file 1). All-donor funding was chosen as the criterion for country stratification, because donor funding has increased relatively more than domestic government funding over the period 2003 to 2009, and is more likely to be allocated to commodities and other variable costs of malaria programmes, as opposed to the more stable cost of human resources, which is usually covered by national governments.

### Insecticide-treated bed net coverage estimates

ITN coverage was assessed as the proportion of households that own one or more ITNs, estimated every year by WHO and partners, for 41 malaria-endemic, low- and middle-income countries in sub-Saharan Africa over the years 2003 to 2010 [3]. The coverage estimation is based on data from nationally representative household surveys (Demographic and Health Surveys, Multiple Indicator Cluster Surveys and Malaria Indicator Surveys), manufacturer reports of ITN procurement and National Malaria Programme reports of ITN distribution [17]. Coverage estimates refer to each country’s population at risk of malaria transmission, assuming that in countries with <100% of the national population living at malaria risk, all available ITNs are owned by households in malarious areas [17]. ITN coverage estimates for national population at risk were not available for countries outside sub-Saharan Africa, where malaria transmission is often focused in certain regions, so that national surveys do not give a meaningful indication of the level of protection in populations actually living in areas with malaria transmission.

### Malaria case and death estimates

Assessments focus on 49 malaria-endemic, low- and middle-income countries outside sub-Saharan Africa, after exclusion of Turkey, Paraguay and Kyrgyzstan, with income levels taken from the World Bank income classification of 2005 [18]. All of these 49 countries were eligible for Global Fund malaria support up to 2010. Turkey, Paraguay and Kyrgyzstan were excluded because they had very few cases throughout the 2000s and small populations at risk, resulting in extremely high levels of funding per person at risk that would have distorted analyses.

WHO’s case estimates for these countries were based on numbers of reported, parasitologically confirmed malaria cases from national health information systems (HIS), adjusted for reporting completeness, the proportion of suspected malaria cases that are parasite-positive, the proportion of confirmed cases due to each *Plasmodium* species, and the extent to which patients use public sector health facilities [3,15,19,20]. Death estimates were derived by multiplying the number of *Plasmodium falciparum* malaria cases estimated for each country by a fixed case fatality rate [3,20]. Case and death estimates were evaluated over the period 2004 to 2010.

For most countries in sub-Saharan Africa, the overall quality of surveillance data did not allow a convincing estimate of total cases to be made from reported cases [3]. For these countries, numbers of cases and deaths are estimated using an epidemiological model in which the trend in each country over time is a function of that country’s increase in household ITN coverage [3]. The dependence of these case and death trend estimates on national ITN coverage trends, itself already considered as progress outcome earlier in the current analysis, precluded an independent assessment of case and death trends in relation to programme funding for the sub-Saharan African countries.

### Malaria funding per case or death averted

Malaria funding allocated (cumulated over 2003–2009) was computed per case or death averted over 2004–2010 for each of the 49 countries outside sub-Saharan Africa. Each country’s time trend in case or death rates over 2004–2010 was estimated in a linear regression, and the predicted value for each of these years compared with case and death rates over 2000–2003 as the baseline, while adjusting for annual population growth. The four-year period 2000 to 2003 (rather than 2003 or 2004 alone) served as the baseline period, because in low-endemic countries outside Africa, ongoing malaria funding serves not only to reduce case and death burden to below 2004 levels, but in the first place to prevent a resurgence to even much higher case and death rates (as seen in 2000 and in the decades before) that would likely reappear if the control stopped.

Country-level programme funding per case or death averted were then summarized for three country groups (tertiles) as the median and interquartile range across the 16 or 17 countries in each group.

## Results

### Malaria financing

**Figure 1:**
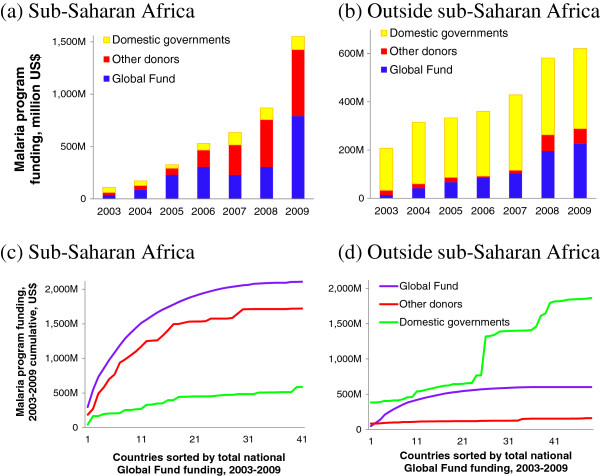
**Malaria programme funding from Global Fund, other donors and domestic governments: aggregated across endemic low- and middle-income countries, by calendar year: (a) 41 countries in sub-Saharan Africa; (b) 49 countries outside sub-Saharan Africa; and by individual country cumulated over 2003 to 2009, with countries sorted by total amount of Global Fund funding over 2003 to 2009: (c) in sub-Saharan Africa; (d) outside sub-Saharan Africa.** Note to Figure 1: Countries with a >0 $ funding level vary among the years; over 2003–2009 summed, out of 90 countries, 16 countries had no Global Fund funding; 14 had no other-donor funding, and eight no domestic funding. From government malaria programme budgets reported to WHO by end-2011 [3], those of Burundi in 2003–2006 and Tanzania in 2008–9 were excluded as they were grossly inconsistent with budgets reported by those countries in neighbouring years.

Total malaria programme funding in the 90 countries increased from US$317 million in 2003 to US$2.2 billion in 2009. The Global Fund, other donors and domestic governments accounted for 47%, 32% and 21%, respectively in 2009 (Figure 1a and b). From 2003 to 2009, Global Fund malaria funding increased 21-fold, other-donor malaria funding increased 14-fold, and domestic government funding increased two-fold. Among non-Global Fund donors, in 2009 the USA Presidential Malaria Initiative contributed around 60% of malaria disbursements in 2009, the World Bank 7%, and the United Kingdom direct bilateral funding through the Department for International Development 6%.

Over 2003 to 2009, funding from all sources combined increased from US$110 million to US$1.55 billion in sub-Saharan Africa (Figure 1a), and from US$207 million to US$620 million outside Africa (Figure 1b). Within sub-Saharan Africa, malaria funding from the Global Fund and other donors was concentrated in a common subset of countries, with half of total Global Fund funding going to only nine countries, and half of other-donor funding going to the same nine countries (Figure 1c). These nine countries were all in east and west Africa (Additional file 1), with stable high rates of malaria case incidence and mortality.

Domestic government funding, in contrast, was concentrated in countries of lower endemicity, with lower case and death rates, in the Americas, Caribbean and Southeast Asia (Figure 1b & d and Additional file 1). Among non-Africa countries, domestic funding increased from $174 million in 2003 to US$ 332 million in 2009 (Figure 1b). Within Africa, domestic funding increased more steeply, from US$ 48 million in 2003 to $125 million in 2009 (Figure 1a).

### Insecticide-treated bed net coverage

**Figure 2:**
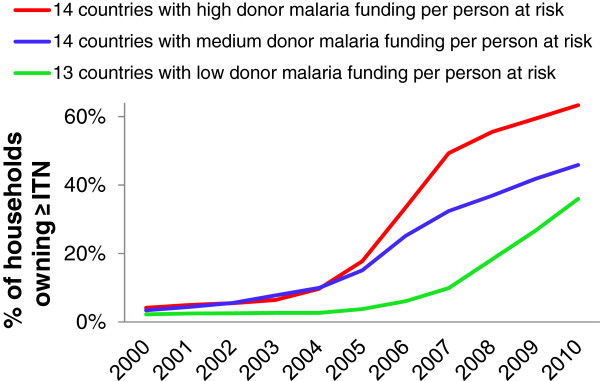
**Progress toward target of universal coverage with ITNs (operationalized as 100% of households owning ≥one ITN [**[3]**]) among 41 malaria-endemic low- and middle-income countries in sub-Saharan Africa, up to 2010, according to the level of countries’ malaria programme funding from donors per person at risk over 2003 to 2009.**

ITN coverage in sub-Saharan Africa increased from 4% in 2003 to 63% in 2010 in the 14 countries with largest donor malaria funding per person at risk over 2003 to 2009, from 3% to 46% in the 14 countries with medium donor funding per person at risk, and from 2% to 36% in the 13 countries with lowest donor malaria funding per person at risk (Figure 2).

### Malaria case and death declines, by level of donor funding

**Figure 3:**
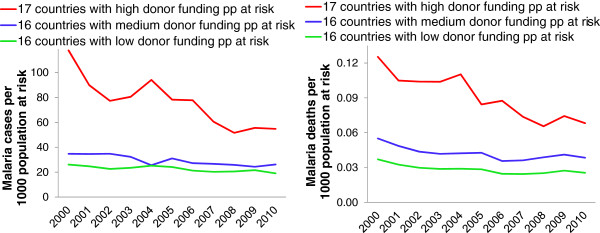
**Progress in reducing (a) malaria case incidence rate, and (b) malaria mortality rate from 2000 to 2010, by countries’ malaria programme funding from all donors combined, over 2003 to 2009 [**[3]**] – among 49 malaria-endemic low- and middle income countries outside sub-Saharan Africa.**

Among 49 countries outside sub-Saharan Africa, the 17 with highest donor malaria funding per person at risk had the highest rates of malaria cases and deaths over 2000–2003, and the largest subsequent declines in these rates, both proportionally and as rate differences compared to the 2000–2003 rates (Figure 3, red lines). Among these 17 countries, overall time trends were driven by the countries with largest numbers of cases and deaths: Papua New Guinea, Cambodia, Sri Lanka, Laos and Timor Leste.

Among the 16 countries with an intermediate level of donor malaria funding per person at risk, an overall slight decline in case and death rates was driven by the relatively high-burden countries: Indonesia, Myanmar and Yemen, where case and death rates were stable; and Afghanistan and Bangladesh, where case and death rates declined (Figure 3, blue lines).

Among the 16 countries with lowest total donor malaria support per person at risk (Figure 3, green lines), the overall case and death rate decline trends were dominated by India, which experienced a very slight decline. Among these countries, Pakistan showed a stable or fluctuating trend, whereas case and death rates declined in most Latin American countries including Brazil.

### Malaria funding per case or death averted

Among countries outside sub-Saharan Africa, programme funding from all sources summed over 2003–2009 per case prevented over 2004–2010 was US$78 (interquartile range US$45−146) in the 17 countries with highest donor funding per person at risk, US$56 (31−260) in the 16 countries with intermediate levels of donor funding per person at risk, and US$5,749 (506−30,227) in the 16 countries with lowest donor malaria support per person at risk (Table 2).

**Table 2:** Estimated cases and deaths prevented over 2004–2010 (relative to 2000–2003 baseline) per dollar total malaria programme funding over 2003–2009, 49 countries outside sub-Saharan Africa

**Countries, outside sub-Saharan Africa**		**17 with highest donor funding per person at risk**	**16 with medium donor funding per person at risk**	**16 with lowest donor funding per person at risk**
Cases prevented over 2004–2010, relative to 2000–3 rates (millions)		6.2	18	20
Deaths prevented over 2004–2010, relative to 2000–3 rates (thousands)		11	15	51
Malaria funding, millions US$ 2003-2009	Global Fund	$ 214	$ 272	$ 115
	Other donors	$ 26	$ 51	$ 85
	Governments domestic	$ 178	$ 243	$ 1,443
	**All sources combined**	**$ 416**	**$ 567**	**$ 1,643**
2003-2009 all-source funding per case prevented, as median across countries		$ 78 (45−146)	$ 56 (31−260)	$ 5,749 (506−30,227)
2003-2009 all-source funding per death prevented, as median across countries		$ 57,654 (24,997−5,484,870)	$ 92,286 (23,391−1,560,418)	$ 3,903,107 (368,839− 19,627,442)

Note: Numbers in brackets are interquartile range across the countries in each group.

Per death prevented over 2004–2010, 2003–2009 funding averaged US$57,654 (24,997−5,484,870) in the 17 countries with highest donor funding per person at risk, US$92,286 (23,391−1,560,418) in the next 16 countries, and US$3,903,107 (368,839− 19,627,442) in the 16 countries with lowest donor malaria support per person at risk. The highest costs per death averted were estimated for countries with very few malaria deaths (Azerbaijan and Honduras in the highest-funding group; Democratic Republic of Korea, the Republic of Korea and Iraq in the medium-funding group; and Mexico and El Salvador in the lowest-funding group), or with an increase in malaria deaths (Timor-Leste and Venezuela in the highest-funding group; Indonesia in the medium-funding group, and Dominican Republic and Costa Rica in the lowest-funding group). The presented upper limits of the interquartile ranges (indicating the cost level that 75% of countries in a funding-level group are below), however illustrate the wide ranges in cost per death averted among all countries.

## Discussion

The synthesis of malaria programme funding and control progress data from countries worldwide confirms that global malaria donor funding is generally well targeted to the countries with highest malaria transmission and case and death rates. Higher donor funding per person at risk was also associated with greater increases in household ITN ownership among African countries, confirming similar observations made earlier [10,15,17,21]. Outside Africa, increased donor funding was associated with larger proportional malaria case and death rate declines.

Outside Africa, all-source programme funding per case averted or death averted tended to be lower in the countries with higher donor malaria support per person at risk (Table 2). These gradients illustrate an overall effective targeting of global donor allocations to countries with highest return on malaria investments. Nevertheless, there was large variation among countries within each group in programme funding per case or death averted, with overlapping ranges across the three tertiles of countries. Apparently, donor allocations partially counterbalance the enormous differences among countries in levels of domestic funding per person at risk and per case or death averted (Figure 1c and d), but they do not completely turn around the cross-country imbalances so as to maximize the possible health impact for available global funding.

The presented ‘costs’ per case or death averted should not be considered as cost-effectiveness ratios, which are typically lower (more favourable) [22,23], for three reasons. First, case/death levels averaged over year 2000−2003 served as the baseline against which cases and deaths averted were calculated; however, in reality, case and death rates in the non-African countries evaluated might well resurge to above 2000−2003 levels if malaria programme funding and control stopped altogether. Second, the time horizon for cases and deaths averted was limited to 2010, without including additional impact achieved over 2011–2012 from the – comparatively large – programme spending in 2009–2010. Third, total funding on all malaria services included case management and programme administration, rather than just the cost of any single intervention.

Although donor malaria funding is generally well targeted to the highest-need countries with lowest ITN coverage and highest case and death rates at baseline, there remain exceptions. As noted before [10,24-26], donor funding per person at risk was very high in some small countries of relatively low malaria burden and high income, including Bhutan, Comoros, Laos, Suriname, and in Africa Sao Tome and Principe and Equatorial Guinea (both excluded from analyses), Gabon, Namibia and Swaziland. In contrast, Burkina Faso, the Democratic Republic of Congo, Mali, Cote d’Ivoire and Nigeria had surprisingly low donor malaria funding relative to their populations at risk.

A full mapping of malaria funding needs and gaps was beyond the scope of the current study, and would have to consider not only actual programme allocations but also countries’ domestic funding capacity. In addition, economies of scale will tend to lower costs per person reached in large-scale, nation-wide programmes in the highest-endemic countries, compared to settings with only focal malaria that require more expensive pre-elimination and elimination strategies [27]. Also, effective progress in control does not necessarily mean that impact would be sustained after lowering the inputs – as experiences with malaria resurgence in Sri Lanka, Madagascar and other countries have shown. According to WHO estimates, overall global funding for malaria control falls short of the global need [3,8,9], and increasing overall malaria funding is equally important as optimizing funding allocations. Nevertheless, the large variation in funding per person at risk (Additional file 1) and the only moderately strong association between funding per case or death averted and level of national donor funding per person at risk (Table 2) suggest a scope for further enhancing value for money in international malaria financing, through more strategic allocation of available funding toward the highest-endemic countries with the highest continued need.

In 2011, the Global Fund revised its eligibility, prioritization and counterpart financing policy. From 2012 onwards all supported countries are required to make a minimum domestic government co-funding contribution, of a proportion increasing with country income and with the years of each grant [28,29]. The increased counterpart financing requirement on upper-middle income countries should result in a gradual shift of portfolio allocations toward lower-income countries. This policy change therefore has the potential to facilitate better targeting of Global Fund funding to benefit countries with the highest continuing need, an approach also emphasized in the Global Fund’s 2012–2016 Strategy ‘*Investing for impact’*[30].

### Limitations

Uncertainties and potential inaccuracies in country ITN coverage and case and death trend estimates [20,21,31] limited the power to assess associations between funding and health impact. The methods for producing estimates of cases and deaths outside of Africa adjust the number of reported cases to take into account the proportion of cases that are not captured by a surveillance system. While helping to make numbers more comparable between countries, and filling gaps where data are missing, the estimates rely on relationships between variables that are uncertain, and draw upon data that may have been imprecisely measured, or measured in previous years and projected forward. Thus estimates of the number of malaria cases or deaths are accompanied by a large degree of uncertainty, and inferences concerning trends are less certain than those made directly from good quality surveillance data. In particular, the number of malaria deaths is estimated by using a fixed case fatality rate which does not take into account varying access to treatment. For sub-Saharan Africa, lack of data on malaria cases and deaths narrowed the analysis to household ownership of ITNs. Although a reasonable predictor of ITN usage [32,33] and associated reductions in under-five mortality, malaria parasitaemia and anaemia [34-36], ownership of one or more ITNs is not a precise indicator of the extent to which universal protection for all household members is achieved.

A further limitation is incompleteness and varying quality of data on domestic government financing. Of the 90 malaria-endemic countries analysed, eight did not report domestic malaria funding to WHO for any of the years 2003 to 2009, and 25 countries did not report domestic funding in 2009. Under-reporting is plausible notably for domestic spending on malaria case management, which is typically not allocated specifically to the malaria programme but rather absorbed within districts’ overall health budgets. While donor funding is sometimes allocated predominantly to commodity needs of malaria programmes [10,15,21], domestic contributions may more often concern the infrastructure, staff and programme management [3]. A recent assessment of global malaria funding over 2006 to 2010 found similar annual funding levels as this study for the Global Fund and other donors, but slightly higher domestic funding – the latter based on government budgets reported by grant recipients to the Global Fund, as opposed to the programme expenditures reported to WHO used here [26].

Because of these imperfections in both national funding and health outcome data, this study assessed their relationship only across groups of countries, and not at the level of individual countries. Importantly, whether in country groups or at the level of individual countries, ‘ecological’ correlations as found in the current observational analysis will not necessarily indicate a true causal impact of programme funding. Among several alternative explanations, programme funding may be associated with increased progress if donors’ funding allocation criteria would effectively prioritize those countries with best programme implementation capacity. Notably the Global Fund selects proposals for grants based on technical soundness and demonstrated effectiveness, followed by performance-based grant renewal [5]. The current low per-person-at-risk donor in some countries in part reflects the challenges that these countries face with effective implementation owing to security concerns, civil strife, limited technical, managerial and strategic planning capacity. Re-allocating funding to such countries may not necessarily yield equally high returns on investment as observed in other countries.

Finally, the study did not consider allocative and technical efficiency in malaria programmes as determinants of progress in control. In reality, not only the funding amount, but also the appropriateness of its allocation across prevention, diagnosis and treatment services, and the − varying − efficiency in delivering these services [37] will influence the relations between funding and health outcome.

To refine these analyses will require improving country-level case and death trend estimates, based on the WHO’s new guidelines for scaling-up malaria diagnosis [38] and for malaria surveillance [39,40], and the ongoing roll-out of parasitological diagnosis notably in African countries [41]. On the financing tracking side, new standards are needed for reporting of national malaria programme financing, to improve quality, transparency, and completeness [42].

## Conclusion

In conclusion, malaria programme financing from the Global Fund and other sources is associated with increased ITN coverage scale-up, a key determinant of malaria burden declines in Africa, and with larger proportional case and death rate declines among countries outside Africa. Achieving MDG6, WHA [43] and RBM targets globally will require accelerated case and death declines, through intensified scale-up of ITN distribution and other key prevention and treatment services. Outside Africa, programme funding per case or death prevented outside Africa tended to be lower in the countries that received higher donor funding per person at risk. The associations shown between funding and impact and the large variations in funding per person at risk suggest opportunities to advance malaria control and maximize the impact by increasing programme funding and strategically allocating available donor funds to countries with the highest continued need.

## Competing interests

The authors’ views do not necessarily represent the decisions, policy or views of Erasmus MC/University Medical Center Rotterdam, the Global Fund to Fight AIDS, Tuberculosis and Malaria, or the World Health Organization. The authors declare that they have no competing interests.

## Authors’ contributions

ELK and RC conceived and designed the study; MH and ELK implemented analyses; ELK, RC and RN drafted the manuscript; all authors contributed to results interpretation and writing of the final manuscript. All authors read and approved the final manuscript.

## Supplementary Material

Additional file 1 ** all-donor and domestic funding national totals and funding per person at risk, estimated malaria cases and deaths rates in 2008 for countries outside sub-Saharan Africa, and population living in households owning ≥one ITN in sub-Saharan African countries in 2008.** Countries sorted by level of all-donor funding per person at risk, separately within sub-Saharan Africa and outside sub-Saharan Africa. Click here for file

## References

[B1] United Nations Department of Economic and Social Affairs The Millennium Development Goals Report 2011 2011 New York City: United Nations

[B2] Roll Back Malaria partnership Refined/updated GMAP Objectives, Targets, Milestones and Priorities Beyond 2011 -- as agreed upon by the RBM Board on 12 June 2011, based on the recommendations of the RBM Task Force on Priorities and Targets Beyond 2011 2011 Geneva: Roll Back Malaria partnership

[B3] World Health Organization World Malaria Report 2011 2011 Geneva: World Health Organization

[B4] World Health Organization World Malaria Report 2012 2012 Geneva: World Health Organization

[B5] The Global Fund to Fight AIDS Tuberculosis and Malaria The Global Fund Results report 2011: Making a difference 2011 Geneva: The Global Fund to Fight AIDS Tuberculosis and Malaria

[B6] The Global Fund to fight AIDS Tuberculosis and Malaria Global Fund-supported programs see strong results amid funding challenges [press release] 2011 Geneva: The Global Fund to Fight AIDS Tuberculosis and Malaria

[B7] Steketee RW Campbell CC Impact of national malaria control scale-up programmes in Africa: magnitude and attribution of effects Malar J 2010 9 299 10.1186/1475-2875-9-299 20979634 PMC2988827

[B8] Kiszewski A Johns B Schapira A Delacollette C Crowell V Tan-Torres T Ameneshewa B Teklehaimanot A Nafo-Traore F Estimated global resources needed to attain international malaria control goals Bull World Health Organ 2007 85 623 630 10.2471/BLT.06.039529 17768521 PMC2636386

[B9] Roll Back Malaria partnership Global malaria action plan for a malaria-free world 2008 Geneva: Roll Back Malaria partnership

[B10] Roll Back Malaria Malaria funding and resource utilization: the first decade of Roll Back Malaria. vol. 10 2010 Geneva: Roll Back Malaria partnership

[B11] The Global Fund to fight AIDS Tuberculosis and Malaria Approved Grant Amounts and Disbursements: Disbursements in detail, Commitments and disbursements -- summary 2012

[B12] United States President's Malaria Initiative Malaria Operational Plans 2011

[B13] Worldbank Data catalog 2010

[B14] Organisation for Economic Co-operation and Development Development Assistance Committee (DAC)'s Creditor Reporting System 2011 Paris: Organisation for Economic Co-operation and Development

[B15] World Health Organization World Malaria Report 2010 2010 Geneva: World Health Organization

[B16] United Nations Dept. of Economic and Social Affairs World population prospects - the 2010 revision population database 2012 New York: United Nations Population Division

[B17] Flaxman AD Fullman N Otten MW Menon M Cibulskis RE Ng M Murray CJL Lim SS Rapid scale-up of insecticide-treated bed net coverage in Africa and its relationship with development assistance for health: a systematic synthesis of supply, distribution and household survey data PLoS Med 2010 17 e1000328 20808957 10.1371/journal.pmed.1000328 PMC2923089

[B18] Worldbank GNI per capita, PPP (current international $) -- World Bank, International Comparison Program database 2012

[B19] World Health Organization World Malaria Report 2008 2008 Geneva: World Health Organization

[B20] Cibulskis R Aregawi M Williams R Otten O Dye C Worldwide incidence of malaria in 2009: estimates, time trends, and a critique of methods PLoS Med 2011 8 e1001142 10.1371/journal.pmed.1001142 22205883 PMC3243721

[B21] World Health Organization World Malaria Report 2009 2009 Geneva: World Health Organization

[B22] Eisele T Larsen DA Walker N Cibulskis R Yukich JO Zikusooka CM Steketee RW Estimates of child deaths prevented from malaria prevention scale-up in Africa 2001–2010 Malar J 2012 93 93 22455864 10.1186/1475-2875-11-93 PMC3350413

[B23] Morel CM Lauer JA Evans DB Cost effectiveness analysis of strategies to combat malaria in developing countries BMJ 2005 331 1299 10.1136/bmj.38639.702384.AE 16282381 PMC1298848

[B24] Institute for Health Metrics and Evaluation Financing global health 2010: development assistance and country spending in economic uncertainty 2010 Seattle, WA: University of Washington

[B25] Snow RW Okiro EA Gething PW Atun R Hay SI Equity and adequacy of international donor assistance for global malaria control: an analysis of populations at risk and external funding commitments Lancet 2010 376 1409 1416 10.1016/S0140-6736(10)61340-2 20889199 PMC2965358

[B26] Pigott DM Atun R Moyes CL Hay SI Gething PW Funding for malaria control 2006–2010: a comprehensive assessment Malar J 2012 11 246 10.1186/1475-2875-11-246 22839432 PMC3444429

[B27] Sabot O Cohen JM Hsiang MS Kahn JG Basu S Tang L Zheng B Gao Q Zou L Tatarsky A Aboobakar S Usas J Barrett S Cohen JL Jamison DT Feachem RG Costs and financial feasibility of malaria elimination Lancet 2010 376 1604 1615 10.1016/S0140-6736(10)61355-4 21035839 PMC3044845

[B28] The Global Fund to Fight AIDS Tuberculosis and Malaria Policy on eligibility criteria, counterpart financing requirements, and prioritization of proposals for funding from the Global Fund 2011 Geneva: Global Fund Twenty-Third Board Meeting

[B29] The Global Fund to fight AIDS Tuberculosis and Malaria Decision Points of the Twenty-Fifth Board Meeting Accra, Ghana, 21–22 November 2011 -- Decision Point GF/B25/DP16: Modification of Grant Renewals and Transition to New Funding 2011 Accra: The Global Fund to fight AIDS Tuberculosis and Malaria

[B30] The Global Fund to fight AIDS Tuberculosis and Malaria The Global Fund Strategy 2012–2016: Investing for Impact 2011 Geneva: The Global Fund to fight AIDS Tuberculosis and Malaria

[B31] Lynch M Korenromp EL Eisele T Newby H Steketee R Kachur P Nahlen B Yoon S MacArthur J Newman R Cibulskis R New global estimates of malaria deaths Lancet 2012 380 559 22883496 10.1016/S0140-6736(12)61320-8

[B32] Korenromp EL Miller J Cibulskis RE Cham MK Alnwick D Dye C Monitoring mosquito net coverage for malaria control in Africa: possession versus use by children under 5 years Trop Med Int Health 2003 8 693 703 10.1046/j.1365-3156.2003.01084.x 12869090

[B33] Miller JM Korenromp EL Nahlen BL Steketee RW Estimating the number of insecticide-treated nets required by African households to reach continent-wide malaria coverage targets JAMA 2007 297 2241 2250 10.1001/jama.297.20.2241 17519414

[B34] Eisele TP Larsen D Steketee R Protective efficacy of interventions for preventing malaria mortality in children in *Plasmodium falciparum* endemic areas / Modeling the impact of scaling up interventions for malaria Int J Epid 2010 39 i88 i101 10.1093/ije/dyq026 PMC2845865 20348132

[B35] Lim SS Fullman N Stokes A Ravishankar N Masiye F Murray CJ Gakidou E Net benefits: a multicountry analysis of observational data examining associations between insecticide-treated mosquito nets and health outcomes PLoS Med 2011 8 e1001091 10.1371/journal.pmed.1001091 21909249 PMC3167799

[B36] Korenromp EL Lives saved from malaria prevention in Africa – evidence to sustain cost-effective gains Malar J 2012 11 94 10.1186/1475-2875-11-94 22455309 PMC3373378

[B37] White MT Conteh L Cibulskis R Ghani AC Costs and cost-effectiveness of malaria control interventions - a systematic review Malar J 2011 10 337 10.1186/1475-2875-10-337 22050911 PMC3229472

[B38] World Health Organization Universal access to malaria diagnostic testing: an operational manual 2011 Geneva: World Health Organization 160

[B39] World Health Organization Disease surveillance for malaria control -- an operational manual 2012 Geneva: World Health Organization

[B40] World Health Organization Disease surveillance for malaria elimination -- an operational manual 2012 Geneva: World Health Organization

[B41] Zhao J Lama M Korenromp EL Aylward P Shargie E Filler S Komatsu R Atun R Adoption of rapid diagnostic tests for the diagnosis of malaria, a preliminary analysis of the Global Fund program data, 2005 to 2010 PLoS Med 2012 7 e43549 10.1371/journal.pone.0043549 PMC3428362 22952703

[B42] Lu C Schneider MT Gubbins P Leach-Kemon K Jamison D Murray CJ Public financing of health in developing countries: a cross-national systematic analysis Lancet 2010 375 1375 1387 10.1016/S0140-6736(10)60233-4 20381856

[B43] World Health Organization Resolution WHA58.2. Malaria control Fifty-eighth World Health Assembly, Geneva, 16–25 May 2005 Volume 1 Resolutions and decisions, and list of participants, WHA58/2005/REC/1 2005 Geneva: World Health Organization 4 7

